# Organ-Protective Effects of Red Wine Extract, Resveratrol, in Oxidative Stress-Mediated Reperfusion Injury

**DOI:** 10.1155/2015/568634

**Published:** 2015-06-16

**Authors:** Fu-Chao Liu, Hsin-I Tsai, Huang-Ping Yu

**Affiliations:** ^1^Department of Anesthesiology, Chang Gung Memorial Hospital, 5 Fu-Shin Street, Kwei-Shan, Taoyuan 333, Taiwan; ^2^College of Medicine, Chang Gung University, Taoyuan, Taiwan

## Abstract

Resveratrol, a polyphenol extracted from red wine, possesses potential antioxidative and anti-inflammatory effects, including the reduction of free radicals and proinflammatory mediators overproduction, the alteration of the expression of adhesion molecules, and the inhibition of neutrophil function. A growing body of evidence indicates that resveratrol plays an important role in reducing organ damage following ischemia- and hemorrhage-induced reperfusion injury. Such protective phenomenon is reported to be implicated in decreasing the formation and reaction of reactive oxygen species and pro-nflammatory cytokines, as well as the mediation of a variety of intracellular signaling pathways, including the nitric oxide synthase, nicotinamide adenine dinucleotide phosphate oxidase, deacetylase sirtuin 1, mitogen-activated protein kinase, peroxisome proliferator-activated receptor-gamma coactivator 1 alpha, hemeoxygenase-1, and estrogen receptor-related pathways. Reperfusion injury is a complex pathophysiological process that involves multiple factors and pathways. The resveratrol is an effective reactive oxygen species scavenger that exhibits an antioxidative property. In this review, the organ-protective effects of resveratrol in oxidative stress-related reperfusion injury will be discussed.

## 1. Introduction

Resveratrol, found in various plants, nuts, and fruits and especially abundant in grapes and red wine, is a naturally occurring plant antibiotic known as phytoalexins [1, 2]. Previous reports have demonstrated the protective effects of resveratrol in different pathological models and experimental conditions [3–6]. Many clinical studies indicate the beneficial effects of resveratrol in human diseases [7–12]. Recent report indicates that intake of a McDonald's meal with red wine could decrease oxidized low density lipoprotein level and increase antioxidative gene expression in healthy human [13]. A growing body of evidence indicates that resveratrol may play potential therapeutic roles in human health by its antioxidant, anti-inflammatory, antiaging, antidiabetic, and apoptotic properties [12, 14–17]. A number of target molecules mediating the abovementioned protective effects of resveratrol have been identified, including the endothelial nitric oxide synthase (eNOS) [18, 19], the mitogen-activated protein kinase (MAPK) [20, 21], the hemeoxygenase-1 (HO-1) [3], the estrogen receptor (ER) [20, 22–24], the histone deacetylase sirtuin 1 (SIRT1) [25–28], the nuclear factor E2-related factor-2 (Nfr2) [3, 29], and nuclear factor-kappa B (NF-*κ*B) [30, 31]. A variety of laboratory and clinical studies also indicate that resveratrol may lead to tissue and organ protective effects against various injuries [6, 19, 32–35]. Ischemia-reperfusion (I/R) injury induces free radical formation and inflammation within hours and results in the excessive production of oxidants and proinflammatory mediators and that play a significant role in the development of multiple organ dysfunctions under those conditions [36–38]. Resveratrol has been suggested as an organ-protective agent to prevent and treat ischemia and shock-like and reperfusion injury due to its antioxidative activities [20, 39–49]. In this review, we summarize the protective effects and possible mechanisms of resveratrol on the preservation of organ function in oxidative stress-mediated I/R injury (Table 1).

## 2. The Organ-Protective Effects of Resveratrol in Ischemia and Reperfusion Injury

### 2.1. Oxidative Stress and Ischemia-Reperfusion Injury

The oxidative stress is still considered to be an important cause of I/R-induced tissue injury. There is a massive increase in oxidants and oxygen radicals during the initiation and progression of I/R injury [50–53]. Ischemia and reperfusion can promote production of ROS, such as superoxide anions (O_2_^−^), hydroxyl free radicals (HO^−^), hydrogen peroxide (H_2_O_2_), and nitric oxide (NO), which is a major factor contributing to I/R-induced organ injury [50, 51, 54, 55]. I/R-induced reactive oxygen species (ROS) formation is the end result of several different oxidant-producing pathways, such as the mitochondria, xanthine oxidase (XO), and nicotinamide adenine dinucleotide phosphate-oxidase (NOX) [50, 52, 56, 57]. Oxygen radicals cause lipid peroxidation that can lead to cell membrane breakdown and mitochondrial damage triggering cell death [21, 58]. NO plays a protective role in I/R injury as increased NO expression can decrease I/R-induced organ injury [59, 60]. In controversy, previous studies have also shown that the inducible NO synthase is upregulated after I/R and can switch from NO to oxygen radical generation under oxidant stress [41, 61]. Oxidative stress could perturb the balance between oxidant and antioxidant status. In most cells, superoxide dismutase (SOD), catalase (CAT), and glutathione peroxidase (GSH-Px) are endogenous free radical scavenging enzymes induced by oxidative stress [62–64]. These antioxidant enzymes play a critical role in the prevention of oxidative damage during ischemia and reperfusion. In addition, the oxidant formation is generated through a series of interacting pathways in various organs and endothelial cells, triggering subsequent leukocyte chemotaxis and inflammation [20, 50, 65]. However, the pathophysiology of I/R injuries has not yet been fully elucidated due to complex interactions and signaling pathways. Previous evidence shows that resveratrol plays an important role organ protection against I/R injury via its antioxidative and anti-inflammatory properties [20, 66, 67]. The protective pathways and mechanisms of resveratrol in ischemia-reperfusion (I/R) will be further discussed in this paper.

### 2.2. The Cardioprotective Effect of Resveratrol in Ischemia-Reperfusion Injury

Myocardial ischemia-reperfusion injury is a complex pathophysiological process that involves various factors and pathways [68–71]. An excess amount of ROS is increased during I/R injury that results in myocardiocyte damage. Resveratrol exerts cardiovascular beneficial effects on atherosclerosis, ventricular arrhythmia, and myocardial I/R injury [4, 72, 73].

Resveratrol could reduce oxidative stress by inhibiting ROS production and has been reported to be a scavenger of hydroxyl, superoxide, metal-induced radicals, and H_2_O_2_ [31, 74–77]. However, the protective effects of resveratrol against oxidative injury are likely to be attributed to the upregulation of the endogenous cellular antioxidant systems rather than its direct ROS scavenging activity. Resveratrol also induces antioxidant enzymes in cardiovascular tissues including SOD, GSH, CAT [72, 74, 78], and NOX [74, 79], all of which are major ROS producing enzymes in the cardiovascular system.

Previous studies have shown that resveratrol-provided cardioprotection is achieved by preserving postischemic ventricular function and reducing myocardial infarct size and cardiomyocytes apoptosis [80]. Pretreatment of rats with resveratrol resulted in cardioprotection in the isolated heart following ischemia and reperfusion [49, 81, 82] and protected neonatal rat cardiomyocytes against anoxia/reoxygenation injury by antiapoptosis [31]. A recent study has shown that resveratrol improved diabetic cardiomyopathy and postischemic ventricular function through regulating myocardial lipoperoxidation and antioxidant enzyme activities [72, 77, 83]. The protective effect is related with an increased activity of peroxidase and superoxide dismutase and a decreased expression of catalase, malondialdehyde (MDA) and isoprostanes [80, 84]. However, MDA is not used as a relaible biomarker of oxidative stress. Instead, isoprostane is considered a specific marker of lipid peroxidation for monitoring oxidative stress [85–87].

NO has been identified as a crucial factor mediating the protective effects of resveratrol [18, 88]. Resveratrol enhances endothelial NO synthase (eNOS) expression in endothelial cells and improves the ventricular function during I/R [18, 88]. SIRT1 has been shown to regulate mammalian genes transcription and has a regulatory function of intracellular signaling in hypoxia or stress [26, 27]. Recent studies indicated that the upregulation of eNOS expression was mediated by SIRT1 [74, 75, 89]. SIRT1 activation may be necessary for the cardioprotective effect, which is mediated by NO signaling [90]. However, other studies have shown that acutely infused resveratrol had no beneficial effect in intestinal ischemia/reperfusion or stroke and it is not mediated by NO elevation [41, 61].

The cardioprotective mechanisms of resveratrol are complex in I/R injury. A previous report demonstrated that there was less myocardial injury and inflammation in Toll-like receptor 4- (TLR4-) deficient mice in I/R. This protective mechanism was possibly associated to the TLR4/nuclear factor-kappa B (NF-*κ*B) signaling pathway [31, 41]. Furthermore, resveratrol attenuates postischemic leukocyte recruitment and subsequent endothelial dysfunction by superoxide-related proinflammatory stimulis, such as hypoxanthine (HX)/XO and platelet-activating factor (PAF) [91].

### 2.3. The Neuroprotective Effect of Resveratrol in Ischemia-Reperfusion Injury

The mechanisms of brain and spinal cord injuries are complex and multifactorial. Oxidative stress has been regarded as important pathogenesis for neurologic damage after cerebral I/R injury. ROS, like superoxide anions, hydroxyl free radicals, hydrogen peroxide, and nitric oxide, are produced during abnormal metabolic reactions or central nervous system activation in I/R [61, 92]. Previous experimental evidence has demonstrated that resveratrol exhibits neuroprotective effect in various cerebral ischemic stroke animal model [3, 93–95]. Treatment with transresveratrol prevented motor impairment, reduced glutathione levels, and also significantly decreased the infarct size after middle cerebral artery occlusion and reperfusion in rat [96]. The neuroprotective effects of resveratrol were shown to be due to its antioxidative and NO promoting properties [48, 92, 97]. Wang and colleagues also showed that resveratrol decreased cerebral microglial activation and delayed neuronal cell death in gerbils, a beneficial effect attributed to its strong antioxidative activity [48]. Previous studies also showed that resveratrol significantly increased the basal levels of adesonine and inosine, inhibited the elevations of hypoxanthine and xanthine levels, remarkably decreased xanthine oxidase activity, and depressed oxidative biomarker (8-isoprostane) levels [84, 92].

Previous studies suggested that the cerebroprotective action of resveratrol could be mediated by both antioxidative and anti-inflammatory effects [98]. Resveratrol treatment decreased oxidative stress and inflammatory markers like isoprostane, tumor necrosis factor-alpha (TNF-*α*), interleukin 6 (IL-6), myeloperoxidase (MPO), and intercellular adhesion molecule 1 (ICAM-1) and increased antioxidative and anti-inflammatory markers like superoxide dismutase, catalase, and interleukin 10 (IL-10) levels in brain I/R injury [84, 99]. Resveratrol pretreatment also reduced astroglial and microglial cells activation by attenuating NF-*κ*B and JNK activation associated with a decrease in inducible nitric oxide synthase (iNOS) and cyclooxygenase-2 (COX-2) production [100]. A recent study has shown that intracortical injection of resveratrol reduced rat cortex infarct volume. This neuroprotective effect was attenuated when resveratrol and a selective estrogen receptor- (ER-) *α* and ER-*β* antagonist injections were given in combination. These results indicated that neuroprotection of resveratrol is mediated via ER-*α* and ER-*β* subtypes [23].

In addition, the resveratrol also has protective effect in spinal cord I/R injury. In a rabbit study, prophylactic use of resveratrol decreased malondialdehyde and myeloperoxidase activity and reduced spinal cord gray matter motor neurons damage following abdominal aorta clamping and reperfusion [46]. Kiziltepe et al. [101] also showed that neuroprotective function of resveratrol in spinal cord I/R injury by scavenging free radicals, inhibiting oxidative stress, and upregulating NO.

### 2.4. The Intestinoprotective Effect of Resveratrol in Ischemia-Reperfusion Injury

Gastrointestinal tract is highly sensitive to I/R injury. Intestinal I/R could trigger the release of oxidants and tissue injurious factors, leading to interstitial edema, microvascular permeability change, vasoregulation impairment, mucosal barrier dysfunction, and inflammatory cell infiltration [65, 102–105].

Resveratrol plays a crucial role in intestinal I/R injuries. Previous study demonstrated that resveratrol exerted its broad spectrum of protective mechanisms through increasing its antioxidative capacity and reducing oxidative status and MPO in intestinal I/R injury [20, 42, 43, 105, 106]. Resveratrol ameliorated the intestinal tissue injury and decreased bacterial translocation in mesenteric lymph nodes via decreased MPO and NO levels and restored SOD activity [43].

Resveratrol at a dose of 0.056 mg/kg significantly decreased the hemoglobin content, the histopathologic score, and tissue myeloperoxidase activity in intestinal I/R injury, without improving the systemic and metabolic parameters [42]. One study showed that intraperitoneal administration of resveratrol reduced excessive NO formation and diminished rat spleen and ileum oxidative damage after hepatic I/R [107]. Furthermore, resveratrol rendered subacute intestinal protection in vivo. Resveratrol significantly ameliorated subacute intestinal I/R injury [41, 42], related to a reduction of NO production and the activation of the SIRT1-NF-*κ*B pathway, which was associated with a decrease in iNOS expression as well as NO production [41]. NO is an important signaling molecule in antioxidative defense mechanisms and resveratrol relieved tissue I/R injuries through an NO-dependent manner [15, 16, 30, 108]. However, its protective or detrimental effect in intestinal I/R injury is still controversial. Some studies showed that an augmented NO production can protect the intestine following I/R injury [12, 109]. Other evidence indicated that a decreased production of NO may have a protective role via with the suppression of inducible NOS in the small intestine I/R [41, 98, 110].

### 2.5. The Renoprotective Effect of Resveratrol in Ischemia-Reperfusion Injury

Renal I/R causes an increase in ROS and isoprostane levels and a decrease of the antioxidant enzyme glutathione in the urological system [111]. Resveratrol may induce the GSH synthesis enzymes and maintain the GSH levels during oxidative stress [45, 112, 113]. It has been shown that resveratrol could maintain antioxidant defenses and reduce the oxidative damage of kidney [45, 113]. Pretreatment with resveratrol prevented the renal I/R-induced lipid peroxidation and protected the depletion of antioxidant enzyme in the renal I/R-treated rats. Moreover, oxidative injury of the kidneys was accompanied by neutrophil infiltration, as evidenced by the elevated tissue MPO levels. In addition, oxidative stress could be involved in renal glomerular lesions caused by a series of proinflammatory mediators, including cytokines and chemokines that lead to the ROS production, leukocyte activation, and glomerular damage [112]. ROS play an important role in the pathologic process of renal ischemia reperfusion injury. Previous study showed that the short-term treatment of resveratrol inhibited renal lipid peroxidation induced by IR. Resveratrol administration decreased renal cortex and medulla damage and reduced the mortality of ischemic rats from 50% to 10% [113].

NO expression is generated in renal tissue and plays an important role in the regulation of renal blood flow and glomerular filtration function. In kidney, resveratrol was found to exert its protective action through the upregulation of NO. Previous studies demonstrated that resveratrol could stimulate NO production during renal I/R [45, 113–116]. Preconditioning and resveratrol treatment also led to a marked increase in NO levels in kidneys and protect renal cells from I/R injury [117]. The protective phenomenon of resveratrol was suggested to be through the NO-dependent mechanism [113]. Another report also evidenced that treatment with L-NAME (an NO synthase inhibitor) attenuated this protection afforded by resveratrol, indicating that resveratrol exerted its protective effect through the release of NO [45].

### 2.6. The Hepatoprotective Effect of Resveratrol in Ischemia-Reperfusion Injury

I/R stimulates the hepatic Kupffer cells and the residing macrophage activation, to generate ROS and proinflammatory cytokines and to upregulate iNOS [60, 118]. The activation of Kupffer cell (KC) with enhanced ROS production and secretion of inflammatory cytokines and proteolytic enzymes is considered to play an important role in liver reperfusion injury [40, 119]. Additionally, the activation of polymorphonuclear leukocytes migration and infiltration in the injury site may enhance the production of inflammatory cytokines, adhesion molecules, and ROS [40, 120].

Previous studies showed that resveratrol reduced liver damage after ischemia-reperfusion. This beneficial effect was due to the replacement of the depleted antioxidant defense system in hepatic I/R injury [121, 122]. Transresveratrol has also been suggested to decrease the superoxide and improve NO bioavailability. Postischemic treatment with lower dose transresveratrol (0.02 mg/kg) reduced TNF-*α*, interleukin 1*β* (IL-1*β*), keratinocyte chemoattractant (KC), and HO-1 hepatic mRNA expression and decreased hepatic neutrophil recruitment [122]. Gedik et al. [121] reported that resveratrol treatment decreased the lipid peroxidation and protected the depletion of antioxidant enzymes (SOD, CAT, and GSH) in rat model of common hepatic artery and portal vein clamping and reperfusion-induced hepatic injury.

### 2.7. The Pulmonoprotective Effect of Resveratrol in Ischemia-Reperfusion Injury

Lung I/R injury occurs in lung transplantation and cardiopulmonary surgery, resulting in an excessive production of reactive ROS [37, 123–125]. The exact mechanism in I/R-induced lung injury is not completely understood. However, an overproduction of ROS such as superoxide and peroxides, activation of macrophages, and infiltration of polymorphonuclear leukocytes are implicated in pulmonary injury [125]. Previous study [89] demonstrated that I/R-induced lung tissue damage was related to pulmonary mitochondrial dysfunction. Peroxisome proliferator-activated receptor-gamma coactivator 1 alpha (PGC-1*α*) was a coactivator controlling aerobic capacity and mitochondrial biogenesis, and an increase in PGC-1*α* mRNA expression was associated with a decreased pulmonary oxidative stress and improved aerobic capacity. Recent study [126] demonstrated that PGC-1*α* mRNA expression in the lungs was markedly improved with resveratrol, providing protection against pulmonary damage induced by contralateral lung I/R injury. Resveratrol treatment also effectively reduced the lipid peroxidation and alveolar neutrophils and maintained mitochondrial homeostasis. In addition, resveratrol could increase mitochondrial activity through upregulating PGC-1*α* and SIRT1 expression [89].

### 2.8. The Reproductive Organs Protective Effect of Resveratrol in Ischemia-Reperfusion Injury

Testicular torsion is urological emergency in which damaged germinal cells may lead to infertility [127–129]. Testicular torsion and detorsion may be regarded as an ischemia/reperfusion (I/R) injury. Oxidative stress is thought to be a major responsible in I/R injury; however, the mechanism involved in testicular injury has not been fully understood. Previous reports demonstrated that ipsilateral testicular torsion could affect the contralateral testis. The injuries caused by I/R were observed in the ipsilateral and contralateral testis [130, 131]. Resveratrol could decrease the cell injury by preventing lipid peroxidation in the cell membrane and DNA damage caused by excessive ROS production [39, 47, 132]. Previous study indicated that treatment with resveratrol improved fertility parameters and contralateral spermatozoid production [133]. A recent report showed that resveratrol pretreatment decreased tissue lipid peroxidation and had a protective effect in the prevention of apoptosis in rat testicular torsion/detorsion (T/D) model [39].

Ovarian torsion is also a gynecological emergency due to the twisting of the adnexa on its ligamentous support. Insufficiency in tissue blood flow due to various reasons such as torsion or embolism leads to ischemia [134, 135]. The levels of ROS and MDA are increased during the reperfusion period following ischemia. It is known that xanthine oxidase is an important source of the ROS production [136, 137] during I/R and GSH is an essential component of the cellular defense mechanism against oxidative cell damage [138, 139]. Hascalik et al. [140] reported that intraperitoneal resveratrol (10 mg/kg) administration reduced the tissue XO products, as well as restored GSH levels, and decreased rat ovarian damage following T/D injury.

### 2.9. The Ophthalmoprotective Effect of Resveratrol in Ischemia-Reperfusion Injury

The retina is a very sensitive neural structure that is easily damaged by free radicals and inflammation following ischemia-reperfusion injury [141, 142]. Retinal ischemia is a common cause of visual loss and impairment. I/R-induced neural injuries are associated with enhanced production of endogenous oxidants such as oxygen free radicals, NO, and calcium [141, 143, 144]. Previous studies showed that resveratrol was capable of crossing the blood-retina barrier and exerting its neuroprotective effects, including cerebral and retinal IR injuries. Vin et al. [145] reported that resveratrol prophylactic treatment attenuated ischemic-induced loss of retinal function and reduced ischemia-mediated thinning of inner retinal layers [145]. Li et al. also showed that pretreatment of resveratrol decreased retinal vascular degeneration by inhibiting endoplasmic reticulum stress in retinal ischemic injury; however, it did not prevent retinal neuronal cell loss [146].

Previous studies showed that matrix metallopeptidase 9 (MMP-9) expression was upregulated during brain ischemia [147] and HO-1 overexpression attenuated retinal cellular damage by intense light exposure [148]. In addition, resveratrol exerted retinal protective effects via modulation of NOS in oxygen-induced retinopathy. Recent study also evidenced that the administration of resveratrol might protect the retina against ischemia by inhibiting iNOS and MMP-9 expression and upregulating HO-1 levels [149].

### 2.10. The Musculoprotective Effect of Resveratrol in Ischemia-Reperfusion Injury

I/R injuries of skeletal muscles are serious clinical problems and are commonly seen in a variety of injuries including traumatic damage, peripheral vascular surgery, plastic surgery, or limb surgery with long time tourniquet application [150–152]. I/R injury of skeletal muscle can increase free radicals production and activate ROS generation, with the ability to produce cell membrane damage and leukocyte infiltration [153–155]. Resveratrol is an effective scavenger of hydroxyl and superoxide and exhibits a protective effect to decrease cell membranes lipid peroxidation and free radicals induced DNA damage [153, 154, 156]. Previous studies have shown that dietary flavonoid resveratrol can protect the skeletal muscle tissue against ischemia and reperfusion injury because of its strong antioxidant properties [157]. Elmali et al. indicated that intraperitoneal resveratrol treatment could exert a protective effect against tourniquet-induced I/R injury in rat gastrocnemius muscle. However, resveratrol not only functioned as an antioxidant but also attenuated the neutrophil infiltration in damaged skeletal muscle [158].

### 2.11. The Vesicoprotective Effect of Resveratrol in Ischemia-Reperfusion Injury

Urinary bladder I/R injury is associated with vascular atherosclerotic disease or pelvic embolization operations [159, 160]. Bladder ischemia could result in detrusor contractility impairment and bladder dysfunction [161, 162]. Previous study showed that I/R injury induced a production of isoprostanes [85, 86] and a decrease in endogenous GSH, as well as an enhanced neutrophil infiltration in rat bladder [162, 163]. However, resveratrol treatment reduced bladder inflammatory cell infiltration, lipid peroxidation, and the myeloperoxidase activity in I/R injury. Resveratrol treatment also reversed the bladder contractile responses to carbachol and prevented oxidative tissue damage following I/R [164].

## 3. The Organ-Protective Effect of Resveratrol in Hemorrhage and Reperfusion Injury

Reperfusion injury after hemorrhage results in an excessive production of oxidants and proinflammatory mediators. The enhanced ROS and proinflammatory cytokines play important factors in the initiation and perpetuation of organ injury [67, 165]. Previous studies have shown that vascular endothelial cell dysfunction can lead to inadequate tissue perfusion, which occurs after hemorrhagic shock and persists despite fluid resuscitation [166, 167]. Oxidative stress and superoxide radical generation are believed to contribute to the pathogenesis of endothelial dysfunction in low-flow states [167, 168]. Endothelial NOX is a major source of ROS of the vasculature, and previous studies have shown that there is a marked increase in NOX-generated ROS by the endothelium under stressful conditions [169, 170]. Elevated ROS is considered a major contributing factor to endothelial dysfunction, and antioxidants have been found to attenuate ROS-induced injuries [168, 169]. Resveratrol has been shown to have broad antioxidative activities in a number of biological systems [170]. Our previous studies have shown that resveratrol prevented hemorrhagic shock-elicited oxidative stress and protected endothelium from subsequent functional damages [168]. The beneficial effects included the suppression of the NOX activity and direct scavenging of ROS. The inhibitory effect of resveratrol on the NOX activity appeared to be mediated through influence of the active NOX enzyme complex assembly in the cell membrane and the cytosol, as evidenced from reduced membrane-bound proteins p22phox and gp91phox and cytosolic protein p47phox [166, 168].

The SIRT1 transcription-modulating proteins showed a fine balance in response to intracellular cues such as hypoxia or stress signals. The beneficial effects of resveratrol mediated by SIRT1 activation can be contributed to by different organs [26, 171, 172]. Studies showed that resveratrol decreased oxidative stress-induce ROS elevation and reduced brain neuron injury by radiation through the activation of SIRT1 [171].

HO-1 appears to act as a protective agent in many organs against insults, such as ischemia and oxidative stress [173, 174]. Previous studies have shown that resveratrol binds and increases the transcriptional activity of ER-*α* and ER-*β*. Resveratrol can modulate HO-1 induction and previous studies have shown that estrogen or flutamide enhances HO-1 expression via ER [174–176]. Our previous studies suggested that the upregulation in HO-1 was associated with the prevention of endothelial dysfunction and the salutary effects of resveratrol on endothelial function, mediated in part by an upregulation of the HO-1-related pathway via ER [165]. The p38 MAPK and Akt have been reported to regulate inflammatory response after trauma hemorrhage [20, 174]. PI3K/Akt pathway is known to play a pivotal role in the ability of neutrophils to undergo chemotaxis. Blockade of Akt activation abolishes the salutary effects of resveratrol in the heart following reperfusion injury [177, 178]. Estrogen-mediated attenuation of the inflammatory response to shock-induced organ injury is abolished by the presence of a p38 MAPK inhibitor (SB-203580) [20, 174]. Previous study also showed that resveratrol administration after hemorrhagic shock upregulated p38 MAPK and Akt expression via HO-1-related pathway [20, 179]. Neutrophils are activated following hemorrhage/reperfusion injury and activated neutrophils appear to infiltrate the injured organs in parallel with increased expression of adhesion molecules on endothelial cells. Upregulation of HO-1 causes a reduction of cytokines, adhesion molecules, chemokines, and neutrophil accumulation and ameliorates organ injury in shock status [20, 180].

## 4. Conclusions

**Figure 1:**
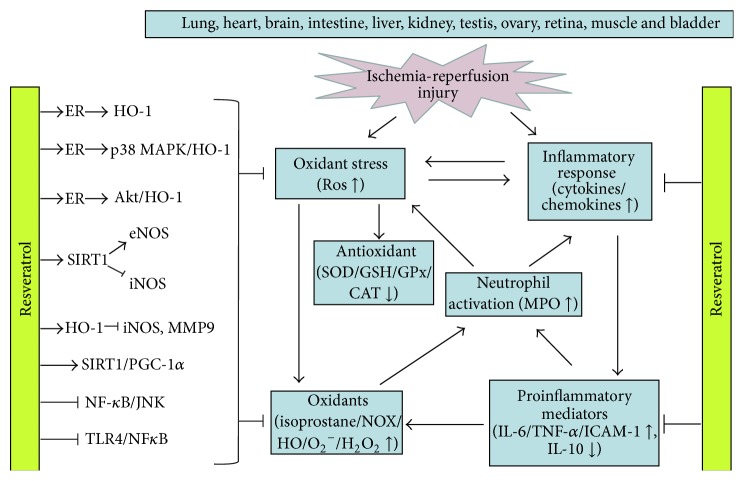
The mechanisms and pathways of resveratrol in oxidative stress-mediated ischemia-reperfusion injury. The protective benefits of resveratrol involved are its scavenging, antioxidant, and anti-inflammatory effect and the signaling mechanisms mediated may be via a variety of intracellular signaling pathways, including upregulation of ER-related MAPK/HO-1 and Sirt1/PGC-1*α* pathway and inhibition of the TLR4 and NF-*κ*B dependent pathway. ROS, reactive oxygen species; ER, estrogen receptor; HO-1, hemeoxygenase 1; SIRT1, sirtuin 1; eNOS, endothelial nitric oxide synthase; iNOS, inducible nitric oxide synthase; TLR4, Toll-like receptor 4; PGC-1*α*, peroxisome proliferator-activated receptor-gamma coactivator 1 alpha; NF-*κ*B, nuclear factor-kappa B; JNK, c-Jun N-terminal kinase; p38 MAPK, p38 mitogen-activated protein kinase; MMP-9, metallopeptidase 9; SOD, superoxide dismutase; CAT, catalase; GSH, glutathione; GSH-Px, glutathione peroxidase (GSH-Px); NOX, NADPH oxidase; XO, xanthine oxidase; O_2_^−^, superoxide anions; HO^−^, hydroxyl free radicals; H_2_O_2_, hydrogen peroxide; TNF-*α*, tumor necrosis factor-alpha; IL-6, interleukin 6; IL-10, interleukin 10; ICAM-1, intercellular adhesion molecule 1; MPO, myeloperoxidase.

Resveratrol has been indicated to have many beneficial effects in various studies and experimental conditions. There is increasing evidence suggesting that resveratrol protects organ function after ischemia or shock-like reperfusion injury. Resveratrol can attenuate organs reperfusion injury through multiple pathways. However, the protective benefits of resveratrol may not simply be attributed by its scavenging, antioxidative, or anti-inflammatory effect. It is implicated that resveratrol is also mediated in part via a variety of intracellular signaling pathways including the regulation of the NOS, HO-1, SIRT1, ER, MAPK, PGC-1*α*, TLR4, and NF-*κ*B (Figure 1). This complex network needs additional elucidation, more experimental studies, and clinical trials. Resveratrol might be a preventive and therapeutic agent to protect reperfusion-induced organ injury in future clinical treatment.

## Figures and Tables

**Table 1:** Protective effects and mechanisms of the resveratrol on different organs in oxidative stress-mediated reperfusion injury.

Species/targets	Model of reperfusion injury	Effective dose	Effects and mechanisms	References
Male Wistar rats rat/heart	Langendorff-perfused mode (ischemia 45 min, reperfusion 10 min).	25 mg/kg (pretreatment 7 days, IP)	MDA↓, CAT↓, peroxidase↑, and SOD↑	[80]

Spraque-Dawley rats/heart	Langendorff-perfused mode (ischemia 60 min, reperfusion 60 min)	20 mg/kg (pretreatment 14 days intragastric tube), 10 *μ*M (30 min before ischemia)	MDA↓, LDH↓, carbonyl↓, and GSH↑	[68]

Male Sprague-Dawley rats/heart	Langendorff-perfused mode (ischemia 30 min, reperfusion 2 h)	10 *μ*M (30 min before ischemia, IV perfused)	MDA↓ and infarct volume↓	[49]

Male Sprague Dawley rats/heart	Langendorff-perfused mode (ischemia 15 min, reperfusion 10 min).	resveratrol 1–100 *μ*M (pretreatment 7 days, IP)	MDA↓ and no improvement in heart function	[82]

Sprague-Dawley/Brain	Right middle cerebral artery occlusion (ischemia 30 min, reperfusion 5.5 h)	0.1–1.0 *μ*M (10 min before ischemia, IV)	Activation of ER-*α* and ER-*β* and infarct volume ↓	[23]

Male Wistar rats/brain	Bilateral common carotid occlusion (occlusion 4 h)	5–30 mg/kg (5 min before reperfusion, IP)	MDA↓, MPO↓, TNF-*α*↓, IL-6↓, ICAM-I↓, Catalase↑, SOD↑, and IL-10↑	[99]

Male Sprague-Dawley rats/brain	Middle cerebral artery occlusion. (occlusion 2 h)	30 mg/kg (pretreatment 7 days, IP)	Adesonine↑, inosine↑, hypoxanthine↓, and xanthine↓	[92]

Male Wistar rats/Brain	Bilateral common carotid occlusion (occlusion 10 min)	30 mg/kg (pretreatment 7 days, IP)	ROS↓, MDA↓, NO↓, and Na^+^K^+^-aTPase↓	[61]

Male Wistar rats/brain	Bilateral common carotid occlusion (occlusion 10 min)	30 mg/kg (pretreatment 7 days, IP)	COX-2↓ and iNOS↓ and NF-kB and JNK activation↓	[100]

Male Sprague-Dawley rats/brain	Middle cerebral artery occlusion (occlusion 30 min)	15 and 30 mg/kg (pretreatment 7 days, IP)	MDA↓, SOD↑, Nrf2↑, HO-1↑, and caspase-3↓	[3]

Mongolian gerbils/brain	Bilateral common carotid occlusion (occlusion 5 min)	30 mg/kg (during occlusion, IP)	Neuronal cell death↓	[48]

Male Wistar rats/Brain	Middle cerebral artery occlusion (occlusion 2 h)	20 mg/kg (pretreatment 21 days, IP)	MDA↓, GSH↑, and infarct volume and motor impairment↓	[96]

Male New Zealand white rabbits/spinal cord	Occlusion of the infrarenal aorta (ischemia 30 min)	1–10 mg/kg (pretreatment 30 minutes, IV)	MDA↓ and NO↑	[101]

Male New Zealand white rabbits/spinal cord	Abdominal aorta clamp (ischemia 30 minute)	100 *μ*g/kg (pretreatment 15 minutes before occluding, IV)	MPO↓, MDA↓, and spinal cord gray matter motor neurons injury↓	[46]

Male Wistar albino rats/intestine	Superior mesenteric artery occlusion (ischemia 60 min, reperfusion 60 min)	15 mg/kg (both before ischemia and before reperfusion, IP)	CAT↑, total antioxidant capacity↑, MPO↓, total oxidative status↓, and oxidative stress index (OSI) ↓	[105]

Male BALB/c mice/intestine	Superior mesenteric artery occlusion (ischemia 1 h, reperfusion 24 h)	50 mg/kg (pretreatment 10 days, PO)	NO↓, iNOS↓, MPO↓, MDA↓, SOD↑, GSH-Px↑, SIRT1↑, and NF-kB↓	[41]

Wistar albino rats/intestine	Superior mesenteric artery occlusion (ischemia 1 hour, reperfusion 24 h)	15 mg/kg (pretreatment 5 days and 15 min before occlusion, IP)	MPO↓, MDA↓, NO↓, and SOD↑	[43]

Male Wistar rat/intestine	Superior mesenteric artery occlusion (ischemia 90 min h, reperfusion 120 min)	0.056 mg/kg (30 min before occlusion, IV)	Intestine damage score↓, MPO↓, and hemoglobin content↓	[42]

Male Wistar albino rats/spleen, ileum	Hepatic artery clamping (ischemia 45 min, reperfusion 30 min)	15 mg/kg (pretreatment 5 days and 15 min before occlusion, IP)	MDA↓, NO↓, and GSH↑	[107]

Male Wistar albino rats/kidney	Right nephrectomy and left renal pedicle clamping (ischemia 45 min, reperfusion 6 h)	30 mg/kg (30 min prior to ischemia and immediately before the reperfusion period, IP)	ROS↓, MDA↓, MPO↓, LDH↓, TNF-*α*↓, SOD↑, and GSH↑	[112]

Male Wistar rats/kidney	Renal pedicles clamping (ischemia 45 min, reperfusion 24 h)	5 mg/kg, (pretrentment 30 minutes before surgery, PO)	NO↑, BUN↓, creatinine↓, SOD↑, GSH↑, and CAT↑	[117]

Male Wistar rats/kidney	Right nephrectomy and left renal pedicle clamping (ischemia 45 min, reperfusion 24 h)	5 mg/kg, (before I/R, PO)	BUN↓, creatinine↓, SOD↑, GSH↑, CAT↑, and NO↑	[45]

Male Wistar rats/kidney	Both renal pedicles cross-clamping (ischemia 40 min, reperfusion 24 h)	0.23 *μ*g/kg (40 min before I/R, IV)	Mortality rate↓, renal damage↓, and NO↑	[113]

Male Sprague-Dawley rat/liver	Clamping the portal vein and hepatic artery (ischemia 1 h, reperfusion 3 h)	0.02 and 0.2 mg/kg (after reperfusion, IV)	IL-1*β*↓, IL-6↓, MPO↓, TNF-*α*↓, KC↓, and HO-1 mRNA↓	[122]

Male Sprague-Dawley rats/liver	Clamping the portal vein and hepatic artery (ischemia 45 min, reperfusion 45 min)	10 mg/kg (15 min before reperfusion, IV)	MDA↓, SOD↑, GSH↑, and CAT↑	[121]

Sprague-Dawley rat/lung	Left hilum (occlusion 60 min)	20 mg/kg (4 days and 15 min before ischemia, PO)	ROS↓, MDA↓, PGC1-*α* mRNA↑, and leukocyte infiltration↓	[126]

Male Sprague-Dawley rat/testis	Left testis torsion/detorsion (ischemia 4 h)	20 mg/kg (30 min before detorsion, IP)	MDA↓, H_2_O_2_↓, and oxidative stress index↓	[39]

Male Wistar rats/testis	Right testis torsion/detorsion (ischemia 4 h)	30 mg/kg (30 min before detorsion, IP; 7 days postoperatively, PO)	Improved contralateral spermatozoid production and some fertility parameters.	[133]

Wistar albino rat/ovary	Right unilateral adnexal torsion/detorsion (torsion 3 h, detorsion 3 h)	10 mg/kg (30 min before detorsion, IP)	MDA↓, XO↓, and GSH↑	[140]

Male Sprague Dawley rats/retinal	Anterior chamber saline bag (intraocular pressure 70–80 mm Hg for 45 min)	30 mg/kg (pretreatment 5 days, IP)	Reduce inner retinal layers thinning	[145]

Male Wistar rats rat/Retinal	Anterior chamber saline bag (intraocular pressure 120 mm Hg for 60 min)	0.5 nmole (pretreatment 15 min, IV)	MMP-9↓, iNOS↓, and HO-1↑	[149]

Male Spraque-Dawley rats/skeletal muscle	Abdominal aorta clamp (ischemia 120 min, reperfusion 60 min)	20 mg/kg (pretreatment for 14 days, gastric tube)	MDA↓, CPK↓, LDH↓, GSH↑ carbonyl↓, and myoglobin↓,	[157]

Sprague-Dawley rats/bladder	Abdominal aorta occlusion (ischemia 60 min, reperfusion 60 min)	10 mg/kg (15 min before I/R, IP)	MPO↓, MDA↓, and GSH↑	[164]

Abbreviations: I/R, ischemia-reperfusion; IP, intraperitonium; IV: intravenous; PO, Orally; MAP mean arterial pressure; ROS, reactive oxygen species; ER, estrogen receptor; HO-1, hemeoxygenase-1; PGC-1*α*, peroxisome proliferator-activated receptor-gamma coactivator 1 alpha; NF-kB, nuclear factor-kappa B; JNK, c-Jun N-terminal kinase; MMP-9, metallopeptidase 9; SOD, superoxide dismutase; CAT, catalase; GSH, glutathione; MDA, malondialdehyde; NOX, nicotinamide adenine dinucleotide phosphate-oxidase; XO, xanthine oxidase; H_2_O_2_, hydrogen peroxide; TNF-*α*, tumor necrosis factor-alpha; IL-6, interleukin 6; IL-10, interleukin 10; ICAM-1, intercellular adhesion molecule 1; MPO, myeloperoxidase; NO, nitric oxide; iNOS, inducible nitric oxide synthase.

## Abbreviations

I/R: Ischemia-reperfusion
IP: Intraperitonium
IV: Intravenous
PO: Orally
MAP: Mean arterial pressure
ROS: Reactive oxygen species
ER: Estrogen receptor
HO-1: Hemeoxygenase-1
PGC-1*α*: Peroxisome proliferator-activated receptor-gamma coactivator 1 alpha
NF-*κ*B: Nuclear factor-kappa B
JNK: c-Jun N-terminal kinase
MMP-9: Metallopeptidase 9
SOD: Superoxide dismutase
CAT: Catalase; GSH: glutathione
NOX: Nicotinamide adenine dinucleotide phosphate-oxidase
XO: Xanthine oxidase
H_2_O_2_: Hydrogen peroxide
TNF-*α*: Tumor necrosis factor-alpha
IL-6: Interleukin 6
IL-10: Interleukin 10
ICAM-1: Intercellular adhesion molecule 1
MPO: Myeloperoxidase
NO: Nitric oxide
iNOS: Inducible nitric oxide synthase.
